# Longitudinal increases of brain metabolite levels in 5-10 year old children

**DOI:** 10.1371/journal.pone.0180973

**Published:** 2017-07-11

**Authors:** Martha J. Holmes, Frances C. Robertson, Francesca Little, Steven R. Randall, Mark F. Cotton, Andre J. W. van der Kouwe, Barbara Laughton, Ernesta M. Meintjes

**Affiliations:** 1 MRC/UCT Medical Imaging Research Unit, Division of Biomedical Engineering, Faculty of Health Sciences, University of Cape Town, Observatory, Cape Town, South Africa; 2 Department of Human Biology, Faculty of Health Sciences, University of Cape Town, Observatory, Cape Town, South Africa; 3 Department of Statistical Sciences, Faculty of Sciences, University of Cape Town, Rondebosch, Cape Town, South Africa; 4 Children’s Infectious Diseases Clinical Research Unit, Department of Paediatrics & Child Health, Faculty of Medicine and Health Sciences, Stellenbosch University, Cape Town, South Africa; 5 A.A. Martinos Center for Biomedical Imaging, Department of Radiology, Massachusetts General Hospital, Charlestown, Massachusetts, United States of America; 6 Department of Radiology, Harvard Medical School, Boston, Massachusetts, United States of America; Indiana University, UNITED STATES

## Abstract

Longitudinal magnetic resonance imaging (MRI) and diffusion tensor imaging (DTI) studies reveal significant changes in brain structure and structural networks that occur together with cognitive and behavioral maturation in childhood. However, the underlying cellular changes accompanying brain maturation are less understood. Examining regional age-related changes in metabolite levels provides insight into the physiology of neurodevelopment. Magnetic resonance spectroscopy (MRS) measures localize brain metabolism. The majority of neuroimaging studies of healthy development are from the developed world. In a longitudinal MRS study of 64 South African children aged 5 to 10 years old (29 female; 29 HIV exposed, uninfected), we examined the age-related trajectories of creatine (Cr+PCr), N-acetyl-aspartate (NAA), the combined NAA+N-acetyl-aspartyl-glutamate (NAAG), choline (GPC+PCh), glutamate (Glu) and the combined Glu+glutamine (Glu+Gln) in voxels within gray and white matter, as well as subcortically in the basal ganglia (BG). In frontal gray matter, we found age-related increases in Cr+PCr, NAA, NAA+NAAG and Glu+Gln levels pointing to synaptic activity likely related to learning. In the BG we observed increased levels of Glu, Glu+Gln and NAA+NAAG with age that point to subcortical synaptic reorganization. In white matter, we found increased levels of Cr+PCr, NAA, NAA+NAAG, Glu and Glu+Gln with age, implicating these metabolites in ongoing myelination. We observed no sex-age or HIV exposure-age interactions, indicating that physiological changes are independent of sex during this time period. The metabolite trajectories presented, therefore, provide a critical benchmark of normal cellular growth for a low socioeconomic pediatric population in the developing world against which pathology and abnormal development may be compared.

## Introduction

Characterizing typical age-related growth patterns in the maturing brain allows for the identification of neurodevelopmental abnormalities or delays from disorders such as attention deficit disorder (ADD) and autism, as well as exposure to toxins or viruses such as HIV. While pediatric neuroimaging studies have described growth trajectories of volumes, cortical thickness, metabolism and brain connectivity, much is still unknown about the physiology underpinning synaptic restructuring and myelination. Studies of metabolites related to synaptic organization and myelination during childhood can provide a deeper understanding of the associated cellular changes.

^1^H magnetic resonance spectroscopy (MRS) measures local brain metabolism, providing information about biochemical aspects of brain maturation. The metabolites typically measured with ^1^H MRS include creatine (Cr+PCr), N-acetyl-aspartate (NAA), choline (GPC+PCh) and glutamate (Glu), which are associated with energy metabolism, neuronal and cellular integrity and neurotransmission. Cross sectional MRS studies have examined age-dependent regional metabolic changes from birth to adulthood, establishing that the most significant changes are in infancy [1–5]. Within studies encompassing ages 5–10 years, some age-related changes in NAA levels as well as NAA/GPC+PCh or NAA/Cr+PCr ratios are reported in gray and white matter [4–8], with one study [5] finding accompanying Cr+PCr levels increasing in gray matter. It is surprising that more metabolites are not implicated in ongoing myelination and synaptic restructuring occurring during this period [9,10]. Possible reasons for the lack of age-related metabolite changes reported include: wide age ranges, small sample sizes, cross-sectional analysis and study of metabolite ratios only. In addition, only one MRS study [6] examined the effects of sex on maturational trajectories, despite sex differences being observed in structural studies [11–14]. Recent neuroimaging studies also found socioeconomic impacts on brain volume development [15,16]. Considering almost all neuroimaging studies on typical brain development in childhood are conducted in the United States, studies of normally developing children from diverse socioeconomic communities in the less developed world are essential.

Neuronal activity modulates how experience and environment shape neural circuit development in childhood [17,18], leading to measurable changes in cognitive and behavioral capabilities. There is growing research relating functional and structural network development to increased cognitive and behavioral abilities [19–21]. The changes in gray matter observed in childhood [11,13,14,22] may be related to synaptic density restructuring [23,24]. Peak synapse formation occurs from about 34 weeks gestation through 2 years of age [9], with the total number of synapses beginning to decrease at puberty [25]. The timeline between 2 years and the onset of puberty, representing when synaptogenesis ends and synaptic pruning starts, is still unclear and is likely to vary regionally [9,10]. Subcortical structures, such as the basal ganglia, are used by the cerebral cortex to process new information. Cognition involves numerous cortical loops through the basal ganglia that involve the prefrontal association cortex and limbic cortex. The basal ganglia (BG) help transform sensory input and cognitive processes into behavior [26]. In white matter, maturation changes are related to ongoing myelination. Even though white matter volume and organization develop well into adulthood [27], the greatest increases in measurements of white matter organization occur by 10 years of age [12,28,29].

To explore cellular changes related to brain maturation in children we describe the longitudinal trajectories of absolute metabolite levels in gray matter, white matter and BG voxels in a typically developing pediatric population aged 5 to 10 years from a low socioeconomic community. From previous studies, we hypothesize age-related increases in NAA levels in all regions, as well as Cr+PCr increases in gray matter. In addition, we investigated the interaction between sex and metabolite levels with age.

The impact of HIV-exposure on trajectories was investigated due to the high rates of HIV infection in the region. Highly successful prevention of mother-to-child transmission (PMTCT) programs have reduced the number of infants acquiring HIV from their mothers. There were 1.3 million women living with HIV in 2013 who gave birth to 199,000 HIV-infected infants and approximately 1 million HIV-exposed, uninfected (HEU) babies [30]. The latter represent a growing population concentrated in Sub-Saharan Africa. Therefore, the local pediatric population includes many HEU children.

## Methods

### Participants

The children presented are a subset of controls from a longitudinal multimodal neuroimaging study addressing the effects of HIV at ages 5, 7 and 9 years. Inclusion criteria for the study were: birth weight > 2000g, no central nervous system problems or dysmorphic syndromes. HIV negative status was confirmed using Roche Amplicor polymerase chain reaction in young infants and negative HIV-1 antibody test in older children. We acquired both magnetic resonance imaging (MRI) and MRS data in 64 children (29 female; 29 HEU). Half of the children received a single scan, twenty children were scanned twice and twelve children had three scans for a total of 108 time points.

HEU children were exposed to treatment for PMTCT, mostly zidovudine antenatally from 28 to 34 weeks and a single dose of nevirapine (NVP) to the mother and zidovudine for a week and a single dose of NVP to the infant.

All children had similar sociodemographic backgrounds and came from low-income communities in Cape Town, South Africa. Table 1 summarizes the available sociodemographic data from a subset of children representative of the sample.

**Table 1:** Summary of sociodemographics for a subset of children scanned.

*Socioeconomic demographics*	
***Housing (n = 55)***	
Housing ^1^	
*Shack*	20%
*Wendy*	11%
*Brick*	69%
Electricity (Yes or No)	Yes (100%)
Water location (Inside or outside)	Inside (65%)
Toilet location (Inside or outside)	Inside (60%)
***Parent income and mortality (n = 55)***	
Deceased parent	
*Father*	16%
*Mother*	2%
Main breadwinner	
*Father*	40%
*Mother*	33%
*Grandparent*	11%
*Sibling*	2%
*Other*	15%
Total monthly income ^2^	
*< $36*	2%
*$36 - $72*	7%
*$72 - $144*	16%
*> $144*	75%
Social grants (Yes or No) ^3^	Yes (75%)
***Parental education***	
Highest maternal education (n = 52)	
*Did not complete through grade 10*	33%
*Grade 10*	52%
*Grade 12*	6%
*Technicon/College diploma*	10%
Highest paternal education (n = 41)	
*Did not complete through grade 10*	43%
*Grade 10*	17%
*Grade 12*	24%
*Technicon/College diploma*	15%

^1^ A shack is defined as an informal dwelling constructed of found materials. A Wendy is a formal housing structure typically made of wood that can be relocated. A brick house is a formal housing structure.

^2^ Amount includes social grants, if received. Amounts converted from South African Rand (ZAR) values to United States dollars (USD) using the current exchange rate of 1 USD = 13.95 ZAR. While no upper income was collected, we note that in a typical recruitment suburb (Khayelitsha) the 2011 census report that 74% of households have a monthly income of $227 or less (www.capetown.gov.za/en/stats/Pages/Census2011).

^3^ The economic qualification for social grants is less than $9894/year ($825/month) for a married couple and $4947/year ($412/month) for a single parent.

### Neuroimaging

Children received neuroimaging without sedation according to protocols approved by the Human Research Ethics Committees of the Universities of Cape Town and Stellenbosch. Parents/guardians provided written informed consent. Children provided oral assent at 5 and 7 years and written assent at 9 years. A senior radiologist reviewed all structural scans, and children with abnormalities were excluded from analysis.

The protocol included a high-resolution T1-weighted 3D EPI-navigated [31] multi echo magnetization prepared rapid gradient echo (MEMPRAGE) [32] acquisition (FOV 224 x 224 mm2, TR 2530 ms, TI 1160 ms, TE’s = 1.53/3.19/4.86/6.53 ms, bandwidth 650Hz/px, 144 sagittal slices, 1.3 x 1.0 x 1.0 mm3) and single voxel ^1^H-MRS in the midfrontal gray matter (MFGM), peritrigonal white matter (PWM) and right BG with a real-time motion and B_0_ corrected [33] point resolved spectroscopy (PRESS; 1.5 x 1.5 x 1.5 cm^3^ voxel; TR 2000 ms, TE 30 ms, 64 averages, 2:16 min) sequence performed on a 3T Allegra Head Scanner (Siemens, Erlangen, Germany) in Cape Town, South Africa.

SPM12 (http://www.fil.ion.ucl.ac.uk/spm) was used for voxel segmentation to determine tissue type percentages for partial volume calculations and water concentration. Frequency/phase correction and eddy current compensation were performed. Absolute metabolite levels (in institutional units) were calculated with LCModel using water scaling [34–36].

**Fig 1:**
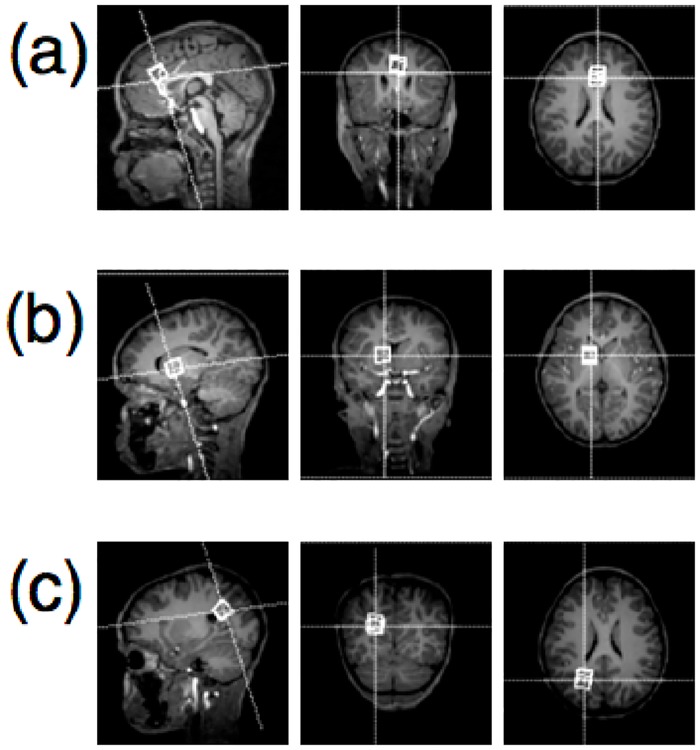
Images of voxel placement. Sagittal, coronal, and axial views are shown from left to right. Location of (a) midfrontal gray matter voxel, (b) basal ganglia voxel, and (c) peritrigonal white matter voxel.

The MFGM voxel (Fig 1a) is comprised primarily of gray matter. The BG voxel (Fig 1b) anatomically represents the frontal limb of the internal capsule and part of the caudate nucleus, putamen and globus pallidus. The PWM voxel (Fig 1c) is mainly white matter. Table 1 summarizes tissue segmentation percentages across ages and voxels.

To ensure quality data, we excluded spectra with full width half maximum (FWHM) > 0.075 and signal-to-noise ratio (SNR) < 7. Within each metabolite, we excluded data points that were more than three standard deviations from the mean for the group.

### Metabolites analyzed

We present developmental trajectories for six metabolites. Total creatine, Cr+PCr, is synthesized in the liver and provides energy to brain cells. NAA is synthesized in neurons and associated with neuronal and axonal integrity [36]. N-acetyl-aspartyl-glutamate (NAAG) is synthesized from NAA and acts as a neuroprotector by modulating the release of glutamate in the synapse [37]. The metabolism of NAA is also considered important in maintaining the integrity of myelinating oligodendrocytes [38]. We report NAA alone and also total NAA levels (NAA+NAAG) which may give insight into the overall state of neuronal integrity and axonal maintenance. Total choline, GPC+PCh, is a marker of cell membrane density and integrity. Glu is the most abundant excitatory neurotransmitter in the brain and is also important in synapse formation, dendrite pruning, cell migration, differentiation and death [39]. Glu is stored as glutamine (Gln) in glia and the cyclical equilibrium of these two metabolites is critical for normal functioning of brain cells [40]. We present trajectories of both Glu and combined Glu+Gln levels.

### Statistical analyses

The R-programming language [41] was used for all statistical analyses. We used a linear mixed effects regression model to study the relationship between age (from 5.1 to 9.5 years) and observed metabolite levels, while accounting for repeated measures in some children.

Sex, ethnicity, and an HIV-exposure variable indicating whether children were exposed to HIV through their mothers, were included as additional covariates in the models examining developmental trajectories of metabolite levels. We included the standard errors of the metabolite estimates to adjust for the differences in measurement accuracy of the different metabolites. The reported standard error estimates, Cramér Rao lower bounds, represent a lower bound of the fitting error by LCModel [33] accounting for both SNR and FWHM parameters to provide an assessment of accuracy of the concentration estimate of each metabolite. In addition, we included gray or white matter tissue percentage of the total tissue (gray + white matter) from segmentation to account for variations in tissue composition. To account for false positives due to hypothesis testing among multiple metabolites and regions, we report adjusted as well as raw p-values. Adjustments for multiple comparisons were performed using the false discovery rate method of Benjamin, Hochberg and Yekutieli [42]. HIV-exposure was included as a covariate due to the high rates of HIV infection in the region. Sex and HIV-exposure interactions with age were examined.

## Results

### Participants

One child was excluded due to an abnormal structural MRI (ex vacuo-dilatation of lateral ventricles) suggestive of a prenatal central nervous system insult. After FWHM and SNR exclusion criteria were applied, the following number of scans were used for analysis in each region: eighty-nine in the MFGM, seventy-seven in the PWM and seventy-five in the BG. Table 2 summarizes the characteristics of children included in analyses in each region.

**Table 2:** Characteristics of children.

	Midfrontal GRAY MATTER	BASAL GANGLIA	Peritrigonal WHITE MATTER
5-year-olds			
*Total number*, *n*	23	23	28
*Age (mean ± standard deviation (sd))*	5.6 ± 0.4 years	5.6 ± 0.4 years	5.6 ± 0.5 years
*Sex*	12 boys/11 girls	12 boys/11 girls	16 boys/12 girls
*Ethnicity*	10 Cape Coloured/13 Xhosa	9 Cape Coloured/14 Xhosa	11 Cape Coloured/17 Xhosa
*HIV exposure*	11 HEU/12 HU	11 HEU/12 HU	16 HEU/12 HU
*SNR (mean ± sd)*	11 ± 2	10 ± 1	10 ± 1
*FWHM (mean ± sd)*	0.03 ± 0.006	0.03 ± 0.005	0.03 ± 0.006
*Tissue fraction (mean ± sd)*	GM: 0.94 ± 0.06	GM: 0.64 ± 0.05	WM: 0.82 ± 0.11
7-year-olds			
*n*	45	36	41
*Age*	7.2 ± 0.1 years	7.2 ± 0.1 years	7.2 ± 0.2 years
*Sex*	25 boys/20 girls	18 boys/18 girls	23 boys/18 girls
*Ethnicity*	9 Cape Coloured/36 Xhosa	7 Cape Coloured/29 Xhosa	7 Cape Coloured/34 Xhosa
*HIV exposure*	18 HEU/27 HU	15 HEU/21 HU	18 HEU/23 HU
*SNR*	10 ± 2	8 ± 1	8 ± 1
*FWHM*	0.03 ± 0.008	0.04 ± 0.008	0.04 ± 0.007
*Tissue fraction*	GM: 0.96 ± 0.02	GM: 0.61 ± 0.06	WM: 0.81 ± 0.14
9-year-olds			
*n*	21	16	8
*Age*	9.2 ± 0.1 years	9.2 ± 0.1 years	9.2 ± 0.1 years
*Sex*	14 boys/7 girls	12 boys/4 girls	7 boys/1 girl
*Ethnicity*	9 Cape Coloured/12 Xhosa	6 Cape Coloured/10 Xhosa	4 Cape Coloured/ 4 Xhosa
*HIV exposure*	10 HEU/11 HU	8 HEU/8 HU	2 HEU/6 HU
*SNR*	9 ± 2	9 ± 1	8 ± 1
*FWHM*	0.04 ± 0.01	0.04 ± 0.008	0.04 ± 0.009
*Tissue fraction*	GM: 0.98 ± 0.02	GM: 0.61 ± 0.06	WM: 0.84 ± 0.06
n, single time point	33	34	40
n, two time points	16	16	14
n, three time points	8	3	3
Total number scans	89	75	77

HEU = HIV-exposed, uninfected. HU = HIV-unexposed, uninfected. GM = Gray matter; WM = White matter; SNR = signal to noise ratio; FWHM = full width half maximum.

The Cape Coloured (mixed ancestry) community is composed primarily of descendants of white European settlers, Malaysian slaves, Khoi-San aboriginals, and black African ancestors.

In the BG, one outlier was excluded in the model for Cr+PCr and for Glu. In the MFGM, one outlier was excluded in the GPC+PCh statistical model.

### Developmental metabolite trajectories

**Fig 2:**
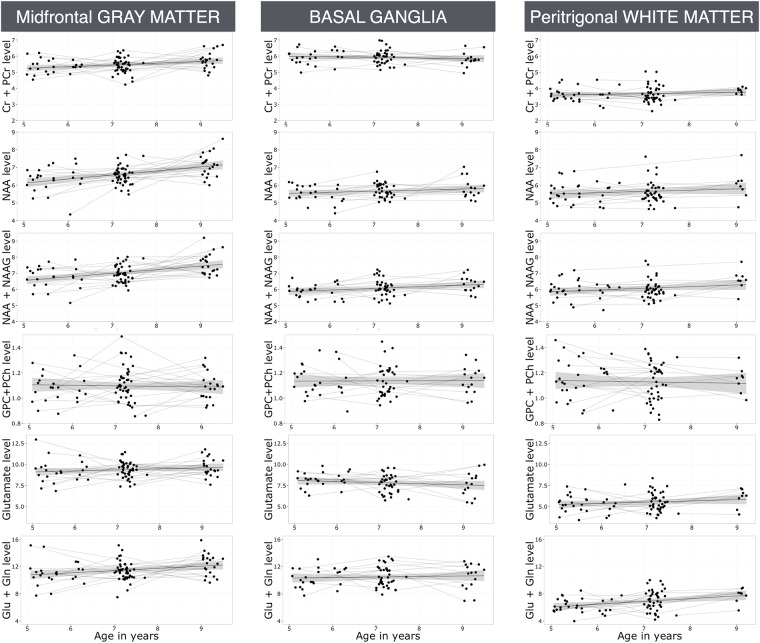
Age-related metabolite trajectories for each region.

Table 3 summarizes the association between age and the metabolite levels in each region. The effect of age is estimated by the slope of a linear regression line and measures the change in the metabolite level associated with a one year increase in age. Plots of their metabolite trajectories in all children are shown for each region in Fig 2.

**Table 3:** Summary of linear mixed effects (LME) model of metabolite levels with age.

	Age effect (standard error)	p-value	Adjusted p-value
Midfrontal gray matter			
*Cr + PCr*	0.13 (0.04)	0.003	0.020
*NAA*	0.20 (0.05)	<0.001	0.004
*NAA + NAAG*	0.17 (0.05)	0.002	0.020
*GPC + PCh*	0.02 (0.01)	0.100	0.400
*Glu*	0.16 (0.09)	0.070	0.300
*Glu + Gln*	0.38 (0.11)	0.002	0.020
Basal Ganglia			
*Cr + PCr*	0.06 (0.04)	0.200	0.600
*NAA*	0.07 (0.04)	0.080	0.300
*NAA + NAAG*	0.16 (0.04)	0.002	0.020
*GPC + PCh*	0.02 (0.009)	0.070	0.300
*Glu*	0.19 (0.07)	0.010	0.060
*Glu + Gln*	0.39 (0.10)	0.007	0.009
Peritrigonal white matter			
*Cr + PCr*	0.10 (0.03)	0.003	0.020
*NAA*	0.14 (0.04)	0.005	0.030
*NAA + NAAG*	0.21 (0.04)	<0.001	0.003
*GPC + PCh*	0.01 (0.01)	0.300	1.000
*Glu*	0.27 (0.06)	<0.001	0.006
*Glu + Gln*	0.41 (0.09)	<0.001	0.004

In addition to age at scan, the LME models include the following covariates: metabolite measurement precision, gray or white matter tissue percentage, sex, ethnicity and HIV exposure (yes or no). Adjusted p-value calculated using the false discovery rate method of Benjamin, Hochberg and Yekutieli.

Fig 2 illustrates the observed mean trajectories of the metabolite levels with increasing age, together with the 95% confidence intervals as indicated by the gray regions.

In the midfrontal gray matter voxel, we found Cr+PCr, NAA, NAA+NAAG and Glu+Gln metabolite levels increased significantly with age (unadjusted and adjusted p-values<0.02. The increase in Glu levels with increasing age were not statistically significant (unadjusted p-value = 0.07, adjusted p-value = 0.400), There was no significant change in metabolite GPC+PCh levels with increasing age (unadjusted and adjusted p-values>0.1).

In the basal ganglia voxel, no significant changes in NAA, Cr+PCr and GPC+PCh levels were found with increasing age (unadjusted and adjusted p-values > 0.07). NAA+NAAG, Glu and Glu+Gln levels showed strong increases with age (unadjusted P<0.01, adjusted p<0.06).

In the white matter voxel, we observed significant increases with age in all metabolite levels (unadjusted and adjusted p-values <0.02) except for GPC+PCh (unadjusted and adjusted p>0.3).

Across regions and metabolites, we observed no significant interactions between sex or HIV-exposure and age (at p < 0.05).

## Discussion

This is the first longitudinal study to investigate metabolite trajectories in three different brain regions of typically developing children from low-income communities. We find age-related changes in metabolite levels–Cr+PCr, NAA, NAA+NAAG, Glu and Glu+Gln–across white and gray matter regions that are likely related to increased synaptic activity and myelination. Our finding of age-related increases in several metabolites, not previously observed, provides a more complex description of the physiology underpinning neurodevelopment.

### Midfrontal gray matter

Between ages 5 and 10 years, neuroimaging studies report increasing total and frontal gray matter volume [11,14,22], increasing and decreasing cortical thickness [43] and changes in functional organization [44]. Previous cross-sectional pediatric studies of metabolism [4,5,7,8] in various gray matter regions report increased NAA levels and NAA/GPC+PCh ratios in this age group, with one study [6] reporting decreased NAA/GPC+PCh. One study [5] reported increased Cr+PCr along with increased NAA levels. These studies implicate changing NAA and Cr+PCr levels alongside structural and functional brain development in gray matter.

Unlike previous studies [4–8], in addition to the expected involvement of NAA and Cr+PCr, our results implicate Glu and Gln in age-related gray matter maturation from ages 5–10 years. The age effects of NAA and the combined NAA+NAAG are similar, suggesting constant NAAG levels. The rise in metabolite level with age of the combined Glu+Gln is larger than Glu, suggesting growth in both glutamine and glutamate levels.

NAA is not only present in neuronal cell bodies, but also in axons, dendrites, and synaptic terminals [36]. The observed increases in NAA and Cr+PCr may be due to dendritic arborizations and the formation of synaptic connections. The observed increase in both Glu and Glu+Gln levels suggests increased demands on the glutamate-glutamine cycle where Glu released from nerve terminals is taken up by surrounding glial cells (astrocytes) and converted to Gln. The growing demand of Glu within the area may be a result of the increased neuronal activity related to learning during the first years of primary school. Additional glutamine reserves in astrocytes are needed to compensate for excess Glu, which can result in unwanted synaptic crosstalk. The increased Cr+PCr may also be a result of the higher neuronal and glial energy demands to support activity. NAA levels reflect not only neuronal and axonal integrity, but also relate to neuronal energetics [36] possibly explaining the similar effect size of the Glu trajectory with age.

Lastly, in MRS studies Cr+PCr is frequently used in metabolite ratios as the denominator value because of its presumed constancy with respect to age. Our results indicate this presumption does not hold in pediatric populations and may produce misleading results.

### Basal ganglia

In this age range, BG volume is observed to both increase [13] and decrease [14,22]. Differences in functional network organization are observed between children aged 7–9 years and young adults, with subcortical regions identified as a major area of difference [44]. In particular, children had more subcortical-cortical connections and these connections were stronger than in adults; the functional development of subcortical connectivity involves changes in wiring and strength of connections [44]. Previous cross-sectional MRS studies [4,5,7] examined similar voxels in the BG in this age range, with only one study [7] reporting an age-related increase in the NAA/GPC+PCh ratio.

In contrast to previous studies, we report increasing levels of Glu, Glu+Gln, NAA and NAA+NAAG. The age-related increase of NAA+NAAG levels with age is about twice that of NAA, implying similar increases in NAAG levels. The age-related rise of both NAA and Glu levels are likely related to the production of NAAG (NAA is combined with Glu to form NAAG). A possible explanation for increased NAAG levels in the BG is its role regulating the release of glutamate and dopamine [37], indicating an age-related increased demand to modulate neurotransmitters during this period. The growth of the combined Glu+Gln levels is greater than Glu pointing to increases in Gln as well as Glu levels. The increased Gln levels may relate to increased demands for Glu to produce NAAG.

For target neurons, Glu is related to two specific outcomes—depolarization and increased cytosolic Ca^2+^. The age-related rise in Glu could also be related to an increase in intracellular Ca^2+^ that triggers biochemical events involved in both functional and structural plasticity of the synapses in childhood [45].

### White matter

Between ages 5 and 10 years, neuroimaging studies have found increasing total white matter volume [11,12,22] and significant changes in measures such as fractional anisotropy describing white matter maturation [12,29], likely related to ongoing myelination. Previous cross-sectional pediatric studies [4–8] in white matter regions report increased NAA and NAAG levels as well as NAA/GPC+PCh ratios.

Contrary to our hypothesis and previous studies, our findings point to the involvement of numerous metabolites—NAA, NAA+NAAG, Cr+PCr, Glu and Glu+Gln—in ongoing myelination from ages 5 to 10 years. Studies have shown both NAA and NAAG play a role in producing and maintaining the myelin sheath, with NAA providing a critical source of acetate for myelin lipid synthesis in oligodendrocytes [36]. Glu has been implicated in myelination, stimulating oligodendrocytes to make myelin protein and begin the process of myelination [45]. Wake et al. (2011) [46] showed that receptors on the cell membrane of oligodendrocytes can identify glutamate released by the axon, which stimulates the creation of signaling domains allowing signals to be passed between oligodendrocytes and axons. Their experiments revealed that without glutamate, myelin production was reduced, highlighting the critical role of glutamate in myelination. The greater increase in Glu+Gln compared to Glu suggests Gln levels are also rising, potentially related to making additional Glu from glutamate-glutamine cycling.

### Conclusion

In infancy, dramatic age-related increases in NAA, Cr+PCr and Glu are observed [2,5]. Combined with our results, these metabolites demonstrate continued alterations due to ongoing brain development. The increases reported here provide evidence of the cellular changes occurring between ages 5 and 10 years during synaptic restructuring and myelination. We find more age-related metabolite level increases during this period than previous studies, likely due to their cross sectional nature and wide age ranges. Given the large individual variability in metabolism during development, both the longitudinal nature of this study and the homogenous sociodemographics of the cohort strengthen the validity of the results.

In summary, we find this is still an important time for influences on learning and development as the 5–10 year old brain continues to undergo growth and development at the cellular level. Therefore, an absence of metabolite level increases during this period may indicate developmental problems or delay. The children in this study were from low-income communities in Cape Town, South Africa, extending knowledge of typical metabolite age-related trajectories to children outside of the developed world. Lastly, these results are particularly relevant as a baseline against which to identify the effects of pathology in pediatric populations.

### Sex and HIV-exposure interactions with age

From ages 5–10 years, the age-related changes in brain metabolism we observed were sex-independent, suggesting similar physiological changes occur across boys and girls even if structural trajectories are different. The lack of HIV-exposure/age interactions in our results implies HEU children are undergoing typical regional development during this period akin to their unexposed peers.

### Limitations

A larger population of children would allow for more statistical power to better study sex-age interactions. We were unable to measure the metabolite levels of NAAG and Gln separately, and have made inferences about their growth from combined measurements.

### Future work

The results presented in this paper are part of an ongoing longitudinal multimodal neuroimaging study in which MRI, DTI and RS-FMRI data were also acquired. Deeper insight into the increases in metabolite levels with age in relation to global changes related to brain maturation may be found by combining these results with morphometric trajectories as well as structural and functional network measures within this cohort. Lastly, this work will be used a benchmark for healthy development against which to compare brain maturation in the HIV-infected children also imaged in the study.

## Supporting information

S1 File Magnetic resonance spectroscopy (MRS) data plus demographic data used in longitudinal analysis for all three regions. The data in the supplemental file includes the absolute metabolite level data for each child in all three regions (basal ganglia, midfrontal gray matter, and peritrigonal white matter) at specific age points. In addition, the covariates used in the model are included for each child, including sex, ethnicity and metabolite level standard error. In addition, we’ve included full width half max (FWHM) and signal to noise ratio (SNR) for all data that met the inclusion criteria. Lastly, we’ve included metabolite ratios (metabolite to Cr+PCr ratio) in addition to the absolute metabolite levels.
(XLSX)
